# Utility of Cadaveric Porcine Heads for Teaching Oral Surgical Procedures in an Australian Dental School: A Pilot Study

**DOI:** 10.3390/jcm13113032

**Published:** 2024-05-22

**Authors:** Jessica Devlin, Yohaann Ghosh, Khilan Shukla, Mark Forwood, Michael Hurrell

**Affiliations:** 1 Oral & Maxillofacial Surgery, Waikato Hospital, Hamilton 3204, New Zealand; 2 School of Medicine & Dentistry, Griffith University, Gold Coast Campus, Southport, QLD 4215, Australia; yohaann.ghosh@lh.org.au (Y.G.); khilan.shukla@my.jcu.edu.au (K.S.); 3 Oral & Maxillofacial Surgery, Royal Brisbane and Women’s Hospital, Bowen Hills, QLD 4006, Australia; 4 School of Medicine & Dentistry, James Cook University, Townsville, QLD 4814, Australia; 5 Anatomy Facility, School of Pharmacy and Medical Sciences, Griffith University, Gold Coast Campus, Southport, QLD 4215, Australia; m.forwood@griffith.edu.au; 6 Oral & Maxillofacial Surgery, Gold Coast University Hospital, Gold Coast, QLD 4215, Australia; michael.hurrell@health.qld.gov.au

**Keywords:** oral and maxillofacial surgery, oral surgery, porcine model, pig head, dental education, mucoperiosteum, surgical flap

## Abstract

**Background/Objectives**: Cadaveric models have traditionally been a mainstay of dental and medical education worldwide since their inception. In Australia, educators at dental schools were among the first to use cadaveric porcine heads in formal teaching in oral surgery. This practice has since fallen out of favour in most modern dental curricula. The aim of this pilot study was to determine the utility of cadaveric porcine models for oral surgery training from a student perspective (Griffith University, Gold Coast, Australia). **Methods**: Thirty participants who were all third-year dental students attended a two-hour session comprising a 30 min lecture followed by a 90 min practical workshop. The lecture outlined the steps and supervision of students during the practical and was provided by a consultant maxillofacial surgeon. At the conclusion of the workshop, participants were asked to anonymously complete a printed questionnaire with eight questions related to their experience. **Results**: Prior to the workshop, two-thirds (61%) of participants felt that they had been taught the surgical procedure for raising mucoperiosteal flaps adequately in their dental school curriculum during their third year, although only 43% of students had assisted specialty residents in raising a mucoperiosteal flap and 14% reported having performed the procedure themselves. Almost all students (96%) agreed that the porcine model was useful for their dental education and that they would practice the exercise using the model again if provided with the opportunity. The questionnaire had a 93.33% completion rate. **Conclusions**: This pilot study indicates that porcine heads present a useful, low-cost adjunct in the learning of basic oral surgical procedures.

## 1. Introduction

Oral surgery is a fundamental component of dental practice, with university curricula worldwide aiming to produce graduates who are competent within the full scope of general dentistry [1,2,3]. In Australia, there is a noticeable trend at recently established dental schools to place an emphasis on theoretical knowledge in oral surgery, without significant complementary practical experience in the discipline [2]. For the majority of modern dental students, practical exposure to oral surgery is limited in the preclinical environment, with students expected to develop skills in exodontia under the supervision of a general dentist [2]. Inadequate exposure to practical oral surgical skills may lead to reduced confidence in oral surgery upon graduation, as well as extended waiting times for specialist referrals [4].

The impact of the COVID-19 pandemic on medical and dental education has also been a source of concern, particularly where electronic and digital formats have superseded or reduced the usual exposure of dental students to oral surgery [5]. Therefore, it is important for dental educators to reflect on which components of online education should be retained, and which areas would benefit from increasing the emphasis on practical skills.

Australian dental educators were among the first reported in the literature to use porcine heads for the formal teaching of oral surgery in dental schools [6]. The low cost, wide abundance, limited regulatory requirements and simple preparation of porcine heads have made them an ideal teaching model for many years [7,8]. Fresh porcine heads are far easier to obtain than fresh human tissue, and in many countries, require far less ethical consideration. However, there are distinct differences between the human and porcine craniofacial complex, most notably the different dentition and thicker soft tissue consistency [9,10]. Porcine heads are frequently used in postgraduate surgical teaching, but there are only limited reports of its use in modern undergraduate (pre-doctoral) dental education [8]. They are commonly used by otolaryngologists, dermatologists, as well as other dental specialties in the training of their residents [10,11,12].

Dental schools commonly face pressures to provide a curriculum for their students that accounts for an ever-expanding knowledge base in dentistry. Condensing this knowledge into a fixed timeframe may have resulted in surgical skill training being neglected at the pre-doctoral level, in favour of greater exposure to advances in restorative and aesthetic procedures [13]. Furthermore, there has been a general trend in dental education where procedural skills are no longer practiced on cadaveric tissue in favour of virtual reality simulation [14,15].

When designing improvements in modern dental curricula, teaching institutions must place great consideration on feedback from the experience of current students [16,17]. The aim of this pilot study was to assess the benefit of using porcine heads as a medium-fidelity model in surgical skill training for third-year undergraduate dental students. To determine the usefulness of this model, students were asked to complete an anonymous questionnaire pertaining to the following: the student’s self-assessment of previous training in dentoalveolar surgery (*Experience*), the usefulness of the porcine model for training (*Teaching*), and how closely the model matched theoretical understanding (*Theory*) and simulated the relevant clinical scenario (*Representation*).

## 2. Materials and Methods

Ethics approval for this study was obtained from the Griffith University, Gold Coast Australia, Human Research Ethics Committee 4 September 2023 (ref. no.: 2023/624). 

Thirty volunteers were recruited for this study. All participants were third-year undergraduate (pre-doctoral equivalent) students in a five-year dentistry program at Griffith University, Gold Coast in Australia. Graduates of the program are eligible for full registration as dentists in Australia and New Zealand, and have reciprocity of training equivalency with Canada. At the time of this study, all students had received one full semester of oral surgery theory education and were familiar with the armamentarium for basic procedures. The third-year cohort was chosen for this activity as they would be expected to perform dental extractions at the university clinic from the commencement of their fourth year.

**Figure 1:**
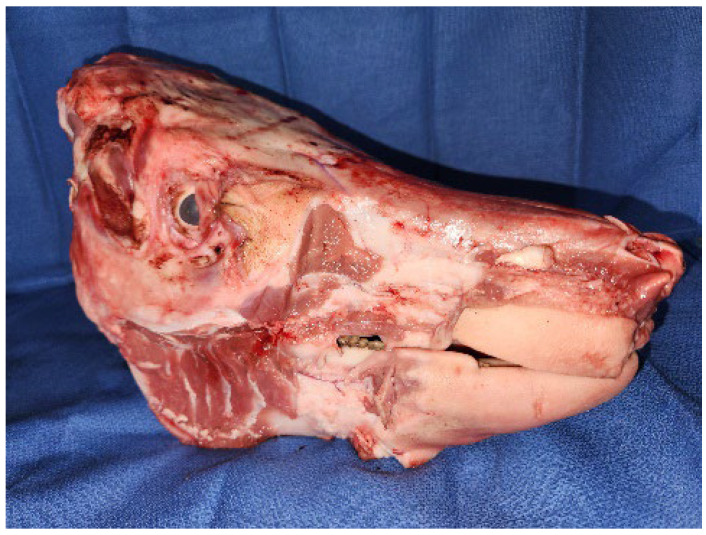
Fresh porcine heads were sourced for an oral surgery workshop from a local abattoir *(Warwick Farms, Warwick, QLD, Australia)*. Excess skin and fat were debulked for ease of transport and storage.

Thirty whole heads were disarticulated at the atlanto-occipital joint from the carcass of commercially farmed pigs (*Sus domesticus*) and processed under Australian food safety standards at a local abattoir (Warwick, QLD, Australia). Each head was purchased for AUD 5.00 (approx. USD 3.30). They were stored at our institution for up to 48 h in a cool room at 4 °C, and set up on blue absorbent pads 1 h prior to teaching, as shown in Figure 1.

Participants attended a single two-hour session that involved a 30 min lecture in basic oral surgery procedures and a 90 min hands-on workshop with porcine heads. The lecture was delivered by a specialist academic oral and maxillofacial surgeon (M.H.), who presented a step-by-step process for the procedures to be completed in the workshop. The hands-on activity involved making a crestal incision through the gingiva of the porcine mandible, raising a mucoperiosteal flap to the level of the molar apices and completing the activity by suturing the flap closed. In addition to a maxillofacial surgeon, students were supervised by two surgical residents and three general dentists (faculty-to-student ratio 1:5).

**Figure 2:**
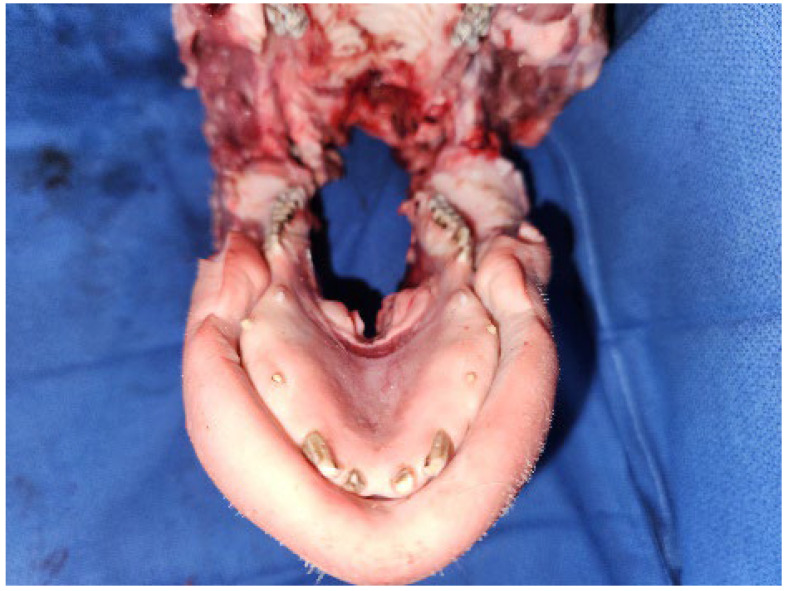
Intra-oral access was achieved by extending incisions through the buccal mucosa bilaterally.

Students were provided with a standard oral surgery armamentarium that included the following: iris scissors, cotton tweezers, Adson forceps, #15 scalpel blade, scalpel handle, Molt periosteal elevator #9, needle holder and 4/0 nylon sutures with a reverse cutting tip. Participants were instructed to expose the porcine heads through the buccal skin and mucosa to provide better access to the oral cavity (Figure 2).

At the conclusion of the workshop, participants were provided with an anonymous questionnaire sheet that included eight prompts presented on a 5-point Likert scale (1: Strongly disagree, 2: Somewhat disagree, 3: Neutral, 4: Somewhat agree, 5: Strongly agree).

The following prompts were included in the questionnaire:

1. Prior to this workshop I have studied the theory of raising mucoperiosteal flaps in my dental school curriculum.
2. Prior to this workshop I have studied the theory of raising mucoperiosteal flaps external to my dental school curriculum.
3. Prior to this workshop I have assisted raising mucoperiosteal flaps.
4. Prior to this workshop I have raised mucoperiosteal flaps.
5. Using the hands-on pig head model helped me relate my previous knowledge and match the practical exercise with theory.
6. The pig head model was representative of what I expect to see clinically when raising mucoperiosteal flaps.
7. Overall, I found that using the pig head model was useful for my dental education.
8. I would practice raising mucoperiosteal flaps in a pig head model again.

Data from all questionnaires were assigned a numerical code and transcribed electronically into Excel 2016 (Microsoft Corporation, Redmond, Washington, DC, USA) for preliminary analysis. All hard copies of the questionnaires were securely destroyed using local document utility services, as per our institutional protocol.

## 3. Results

Of the thirty participants, twenty-eight completed the questionnaire, and two participants returned an incomplete form to the response collection box (93.33% completion rate). As all responses were anonymous, participants who did not complete the questionnaire were unable to be followed up.

**Figure 3:**
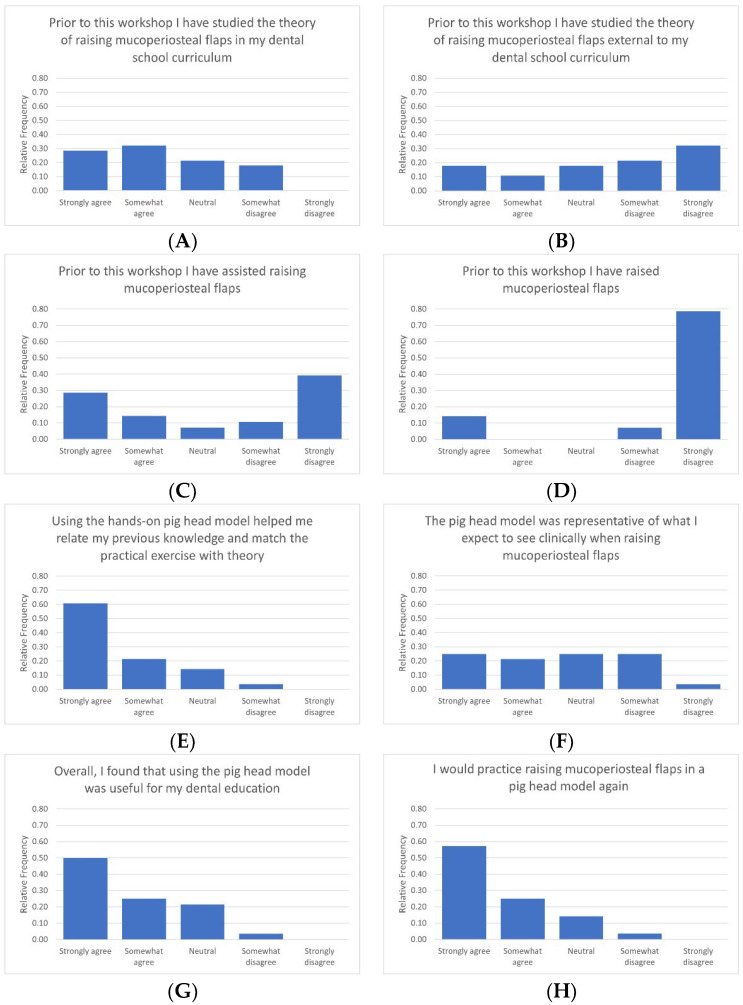
Survey responses of the participants post completion of the session. (**A**) Theoretical knowledge of surgical technique for raising mucoperiosteal flap previously delivered in the school curriculum. (**B**) Theoretical knowledge of surgical technique for raising mucoperiosteal flap previously studied outside of the school curriculum. (**C**) Previous experience in assisting in raising the mucoperiosteal flap. (**D**) Previous experience in the raising the mucoperiosteal flap as the primary operator. (**E**) Perceived reinforcement of theoretical knowledge with practical application. (**F**) Perceived similarity between the pig head model and application to a clinical setting. (**G**) Perceived usefulness of the session. (**H**) Interest in repeating the exercise.

The results from the questionnaire are summarised in Figure 3. Prior to the workshop, two-thirds (61%) of participants felt that they had been taught the surgical procedure for raising mucoperiosteal flaps adequately in their dental school curriculum and only one-third (29%) of students reported studying the theory external to the dental school curriculum. Less than half of the students (43%) reported having experience in assisting specialty residents raising a mucoperiosteal flap, and only 14% reported having performed the procedure themselves.

Seventy percent of the students agreed that the model was representative of what they have or expected to see clinically, and only 4% of students felt that the session did not help in relating prior theoretical knowledge. Almost all students (96%) agreed that the porcine model was useful for their dental education and that they would practice the exercise using the model again if provided with the opportunity (96%).

## 4. Discussion

The competence of dental students in oral surgery requires a balance between theoretical understanding and clinical practice [2]. Intentional practice on a representative model is a critical aspect of developing expertise in any surgical discipline and forms the basis for many surgical training programs [18]. The opportunity to experience haptic feedback allows for clinicians to develop confidence performing the procedure, as well as understanding its indications and contraindications, and the purpose of each instrument in their armamentarium. It is equally important that the development of practical skills is conducted in an environment and on a model that is perceived to be useful [17]. While other models such as periodontal typodonts and virtual simulation exist as alternatives to porcine tissue, they do not provide haptic feedback when raising the periosteum to the same level [14,19]. We did not use human cadaveric material due to the extensive and expensive logistical and ethical limitations of using fresh tissue in our jurisdiction. On the other hand, using pig heads from a local commercial abattoir posed far less ethical restrictions under our institutional protocol.

Dental students in their third year of study at Griffith University are exposed to pre-recorded lectures on the theory of oral surgery, as well as an explanation on how to use basic surgical instruments. The results of the questionnaire indicated that 70% of the students were in a phase where the opportunity for practical skill training was able to complement their theoretical understanding in raising an oral mucoperiosteal flap. As a medium-fidelity model, porcine heads are not solely adequate for oral surgery training but do provide a method that preclinical students find useful for developing practical skills in oral surgery. Within the cohort, there was a distinct group (29%) that benefited the most from this activity—those who had completed theoretical training but had not yet assisted a surgeon during their mandatory placement hours. During their mandatory assisting hours, it is possible that an oral surgeon had encouraged a student to perform part of the procedure, and this could account for outliers with respect to experience, despite all students being in the third year of their pre-doctoral program.

Goss et al. [2] reported that fewer than half of the dental schools throughout Australia and New Zealand taught dentoalveolar surgery in dedicated oral and maxillofacial surgery (OMS) clinics under direct supervision of surgically trained staff. They found that while all schools provided education regarding the removal of impacted teeth, none expected students to be competent in doing so upon graduation. The opportunity to attend an OMS-directed workshop in practical oral surgical skills may therefore lead to improved understanding and confidence of dental students upon graduation.

This pilot study demonstrated that 96% of students responded favourably to the use of a porcine model in the development of their learning. The modality of porcine heads may also lead to improved patient outcomes, as students would have better technical skills by practicing on a medium-fidelity-representation model prior to treating human patients. The authors hope that this pilot study promotes the implementation of further practical wet-lab workshops in the modern dental curriculum, with a focus on providing pre-clinical students with adequate training in practical skills.

## 5. Conclusions

Overall, third-year Australian dental students at Griffith University found that using porcine heads was a useful adjunct to learning basic oral surgical procedures. Compared to other training models, porcine heads are a relatively low-cost investment for educators and present a unique opportunity for dental students to seek feedback on their surgical skills prior to treating patients. We hope that this pilot study encourages more dental schools to incorporate practical surgical skills training in their curriculum, with a focus on improving patient outcomes and the confidence of their students upon graduation.

## Data Availability

The raw data supporting the conclusions of this article will be made available by the authors on request.
